# Antifungal prophylaxis with nebulized amphotericin-B in solid-organ transplant recipients with severe COVID-19: a retrospective observational study

**DOI:** 10.3389/fcimb.2023.1165236

**Published:** 2023-04-27

**Authors:** Alexander Rombauts, Marta Bodro, Victor Daniel Gumucio, Irene Carbonell, Àlex Favà, Laura Lladó, José González-Costello, Federico Oppenheimer, María Ángeles Castel-Lavilla, Oscar Len, Ester Marquez-Algaba, Xavier Nuvials-Casals, Daniel Martínez González, Judith Sacanell Lacasa, Jordi Carratalà, Nuría Sabé

**Affiliations:** ^1^ Department of Infectious Diseases, Hospital Universitario de Bellvitge, L’Hospitalet de Llobregat, Barcelona, Spain; ^2^ Department of Infectious Diseases, Hospital Clínic de Barcelona, Barcelona, Spain; ^3^ Department of Intensive Care Medicine, Hospital Universitario de Bellvitge, L’Hospitalet de Llobregat, Barcelona, Spain; ^4^ Renal Transplant Unit, Hospital Universitario de Bellvitge, L’Hospitalet de Llobregat, Barcelona, Spain; ^5^ Liver Transplant Unit, Hospital Universitario de Bellvitge, L’Hospitalet de Llobregat, Barcelona, Spain; ^6^ Heart Transplant Unit, Hospital Universitario de Bellvitge, L’Hospitalet de Llobregat, Barcelona, Spain; ^7^ Renal Transplant Unit, Hospital Clínic de Barcelona, Barcelona, Spain; ^8^ Heart Transplant Unit, Hospital Clínic de Barcelona, Barcelona, Spain; ^9^ Department of Infectious Diseases, Hospital Universitari Vall d'Hebron, Barcelona, Spain; ^10^ Department of Medicine, Universitat Autònoma de Barcelona, Barcelona, Spain; ^11^ Centro de Investigación Biomédica en Red de Enfermedades Infecciosas (CIBERINFEC), Instituto de Salud Carlos III, Madrid, Spain; ^12^ Department of Intensive Care Medicine, Hospital Universitari Vall d'Hebron, Barcelona, Spain; ^13^ Bellvitge Biomedical Research Institute (IDIBELL), L´Hospitalet de Llobregat, Barcelona, Spain

**Keywords:** COVID-19, SARS-CoV-2, solid-organ transplant recipients, amphotericin-B, prophylaxis, Aspergillus spp., CAPA, Aspergillosis

## Abstract

COVID-19-associated pulmonary aspergillosis (CAPA) has emerged as a frequent complication in the intensive care unit (ICU). However, little is known about this life-threatening fungal superinfection in solid organ transplant recipients (SOTRs), including whether targeted anti-mold prophylaxis might be justified in this immunosuppressed population. We performed a multicentric observational retrospective study of all consecutive ICU-admitted COVID-19 SOTRs between August 1, 2020 and December 31, 2021. SOTRs receiving antifungal prophylaxis with nebulized amphotericin-B were compared with those without prophylaxis. CAPA was defined according the ECMM/ISHAM criteria. Sixty-four SOTRs were admitted to ICU for COVID-19 during the study period. One patient received antifungal prophylaxis with isavuconazole and was excluded from the analysis. Of the remaining 63 SOTRs, nineteen (30.2%) received anti-mold prophylaxis with nebulized amphotericin-B. Ten SOTRs who did not receive prophylaxis developed pulmonary mold infections (nine CAPA and one mucormycosis) compared with one who received nebulized amphotericin-B (22.7% vs 5.3%; risk ratio 0.23; 95%CI 0.032-1.68), but with no differences in survival. No severe adverse events related to nebulized amphotericin-B were recorded. SOTRs admitted to ICU with COVID-19 are at high risk for CAPA. However, nebulized amphotericin-B is safe and might reduce the incidence of CAPA in this high-risk population. A randomized clinical trial to confirm these findings is warranted.

## 1 Introduction

Several studies have shown an association between invasive pulmonary aspergillosis and viral infections, such as cytomegalovirus disease (Singh and Husain, 2009; Kuo et al., 2022) and influenza (Schauwvlieghe et al., 2018; Verweij et al., 2020). Worryingly, recent large cohort studies of COVID-19-associated pulmonary aspergillosis (CAPA) in patients admitted to the intensive care unit (ICU) have raised concerns about this superinfection. Indeed, this life-threatening secondary infection appears to develop in approximately 18-19% of patients with critical COVID-19 and significantly increases mortality (Bartoletti et al., 2021; Dupont et al., 2021; Xu et al., 2021; Gangneux et al., 2022; Prattes et al., 2022).

Solid organ transplant recipients (SOTRs) are at increased risk of severe COVID-19 (Trapani et al., 2021; Maggiore et al., 2022), and ICU admission than the general population (Gatti et al., 2022). Because of baseline immunosuppression, CAPA is likely to be more frequent in SOTRs than in the general population admitted to ICU for severe COVID-19. Unfortunately, data about CAPA in SOTRs is extremely scarce. Indeed, when combining data from three of the largest studies published to date, only 44 of the 1209 included patients were SOTRs (Bartoletti et al., 2021; Gangneux et al., 2022; Prattes et al., 2022). Of concern, however, a staggering 31,8% (14 out of 44) had CAPA (Bartoletti et al., 2021; Gangneux et al., 2022; Prattes et al., 2022).

Antifungal prophylaxis might be justified for high-risk groups due to the incidence and mortality of CAPA in ICUs. Unfortunately, antifungal prophylaxis in SOTRs is not without challenges. Drug-drug interactions exist between triazoles and the most common immunosuppressants (calcineurin inhibitors and mammalian target of rapamycin [mTOR] inhibitors) (Lempers et al., 2015). Echinocandins have poor activity against molds with higher rates of breakthrough infections (Gomes et al., 2014; Lionakis et al., 2018), while the use of intravenous amphotericin-B is limited by the risk of renal injury and the potential for drug-drug interactions (Trejtnar et al., 2014). In this scenario, nebulized amphotericin-B, ensuring high alveolar concentrations (Fauvel et al., 2012) with limited systemic exposure, seems an attractive choice. Clinical studies among lung (Peghin et al., 2016; Huggins et al., 2022) and cardiac transplant recipients (Paniagua Martin et al., 2010), plus neutropenic hematological patients (Rijnders et al., 2008), show that this approach has high efficacy and causes few adverse events.

In this retrospective study, we aimed to evaluate the impact of antifungal prophylaxis with nebulized amphotericin-B in SOTRs admitted to ICU for COVID-19.

## 2 Material and methods

### 2.1 Study design, setting, patients and definitions

We conducted a multicenter, real-world, retrospective cohort study among adult (age ≥ 18 years) SOTRs admitted with COVID-19 to ICUs in Barcelona between August 1, 2020 and December 31, 2021. Three university hospitals with transplant programs took part (Hospital Universitari de Bellvitge, Hospital Clínic de Barcelona and Hospital Universitari Vall d’Hebron) and collected the following data from the electronic health record during hospitalization: demographic, comorbidities, symptoms, oxygen requirements, radiological features, treatments, microbiological and analytic parameters, and clinical courses. COVID-19 associated aspergillosis was defined according to the European Confederation of Medical Mycology (ECMM)/International Society for Human and Animal Mycoses (ISHAM) criteria (Koehler et al., 2021). None of the studies sites reported environmental issues which might influence the development of aspergillosis.

### 2.2 Antifungal prophylaxis regimen

All three centers employed the same antifungal prophylaxis regimen, giving a 24 mg dose of nebulized liposomal amphotericin-B three times a week. Mechanically ventilated patients received 6 mg of nebulized amphotericin-B desoxycholate every 8 hours. See **Supplementary Material** for more detail. We excluded participants if they received a different antifungal prophylaxis and patients of the first wave of the COVID-19 pandemic to limit heterogeneity. After witnessing several CAPA cases in COVID-19 ICU admitted SOTRs in the first year of the pandemic, the infectious diseases consultants encouraged the use of prophylaxis with nebulized amphotericin-B, although the final decision on initiating prophylaxis remained with the treating intensivist.

### 2.3 Statistical analysis

Descriptive statistical analysis was performed for all study variables. Data are reported as means and standard deviations or as the percentage of patients with data available for a given variable. We then compared patients who did and did not receive nebulized amphotericin-B. To identify risk factors, we also compared patients with and without CAPA. Chi-squared tests were used to assess categorical variables and student t-tests or Mann–Whitney *U* tests were used to assess quantitative variables.

### 2.4 Ethics

The study protocol was approved by the Clinical Research Ethics Committee of the Bellvitge University Hospital (EOM039/21) and the procedures were conducted in accordance with the ethical standards of the Helsinki Declaration. The need for informed consent was waived because of the retrospective observational nature of the study and the use of de-identified anonymous clinical data.

## 3 Results

During the study period, 64 SOTRs were admitted to ICUs with COVID-19 pneumonia. Of the 20 patients, (31.3%) who received antifungal prophylaxis, 19 received nebulized amphotericin-B, while one patient receiving isavuconazole prophylaxis was excluded from the analysis.

The analyzed sample included 63 SOTRs (mean age, 62.1 ± 11.7 years), 49 renal, 6 lung, 4 heart, 3 liver, and 1 pancreas/kidney recipients, of which 16 (25.4%) had undergone transplantation in the 6 months before ICU admission. Maintenance immunosuppression typically included tacrolimus (90.5%) with mycophenolate (85.7%), whereas the acute treatment of COVID-19 included corticosteroids (96.8%), tocilizumab (25.4%), and remdesivir (9.5%), with 77.7% requiring invasive mechanical ventilation. The in-hospital mortality rate was 57.1%.

The mean time to CAPA diagnosis after ICU admission was 6.8 ± 5.8 days, with intensivists diagnosing 1 possible and 9 probable cases of aspergillosis, together with 1 case of pulmonary mucormycosis (17.4%). The ECMM/ISHAM criteria used to establish pulmonary aspergillosis can be found in the Supplementary Material (**Table S1**). The characteristics of patients with and without aspergillosis can be found in the Supplementary Material (**Table S2**). Univariate analysis revealed that SARS-CoV-2 vaccination (41.5% vs 80%; p=0.025) was associated with CAPA and a trend for higher CAPA risk among SOTRs treated with tocilizumab (50%) compared to those not treated with tocilizumab (50% vs 20.8%; p=0.051). Patients with and without CAPA did not differ by ICU length of stay, duration of invasive mechanical ventilation, or in-hospital mortality.

The characteristics of patients with and without prophylaxis are shown in **Table 1**. The mean duration of prophylaxis was of 21.1 days (SD 15.2). Of note, 10 patients (22.7%) in the non-prophylaxis group developed a pulmonary fungal infection (9 CAPA, 1 mucormycosis) compared to 1 patient (5.3%) in the prophylaxis group (risk ratio 0.23; 95%CI 0.032–1.68).

**Table 1:** Comparison between SOTRs with and without anti-mold prophylaxis at ICU admission for COVID-19.

Variable	Anti-mold prophylaxis		p value	
	Without, n=44 (%)	With, n=19 (%)^a^		
Patients characteristics				
Age, years; mean (SD)	61.3 (11.85)	64.25 (11.4)	ns	
Female (n=16)	14 (31.8)	2 (10.5)	ns	
Active smoker (n=2)	2 (4.5%)	0 (0)	ns	
High blood pressure (n=54)	41 (93.2)	13 (68.4)	0.01	
Chronic renal failure (creatinine > 136 μmol/L) (n=41)	33 (75)	8 (42.1)	0.012	
Diabetes mellitus (n=26)	19 (43.2)	7 (36.8)	ns	
Preexisting heart diseases (n=25)	17 (38.6)	8 (42.1)	ns	
BMI >30 (n=15)	9 (20.5)	6 (31.6)	ns	
Preexisting lung diseases (n=13)	5 (11.4)	8 (42.1)	0.006	
Chronic hepatopathy (n=4)	2 (4.5)	2 (10.5)	ns	
Kidney transplant^b^ (n=50)	38 (86.4)	12 (63.2)	0.037	
Lung transplant (n=6)	0 (0)	6 (31.6)	<0.001	
Heart transplant (n=4)	4 (9.1)	0 (0)	ns	
Liver transplant (n=3)	2 (4.5)	1 (5.3)	ns	
Recent SOTR (<6 months) (n=16)	10 (22.7)	6 (31.6)	ns	
Hepatitis C infection (n=2)	2 (4.5)	0 (0)	ns	
CMV viremia (<3 months) (n=7)	1 (2.3)	6 (31.6)	0.001	
Transplant rejection (<3 months) (n=6)	2 (4.5)	4 (21.1)	0.041	
Corticosteroid bolus (<3 months) (n=9)	4 (9.1)	5 (26.3)	0.073	
ATG (<6 months) (n=2)	1 (2.3)	1 (5.3)	ns	
SARS-CoV-2 vaccination (n=30)	19 (43.2)	11 (57.9)	ns	
Immunosuppressants at hospital admission				
Cyclosporine (n=2)	2 (4.5)	0 (0)	ns	
Tacrolimus (n=57)	39 (88.6)	18 (94.7)	ns	
Sirolimus (n=2)	1 (2.3)	1 (5.3)	ns	
Everolimus (n=2)	2 (4.5)	0 (0)	ns	
Mycophenolate mofetil (n=54)	38 (86.4)	16 (84.2)	ns	
Immunosuppressants at ICU admission				
Calcineurin inhibitors^c^ (n=42)	28 (63.6)	14 (73.7)	ns	
mTOR inhibitor (n=2)	2 (4.5)	0 (0)	ns	
Mycophenolate mofetil (n=9)	6 (13.6)	3 (15.8)	ns	
COVID-19 Treatment				
Tocilizumab (n=16)	14 (31.8)	2 (10.5)	p=0.075	
Remdesivir (n=6)	4 (9.1)	2 (10.5)	ns	
Corticosteroids (n=61)	43 (97.7)	18 (94.7)	ns	
High-dose corticosteroids^d^ for COVID-19 pneumonia (n=11)	4 (9.1)	7 (36.8)	p=0.008	
Clinical course and outcomes				
Invasive mechanical ventilation (n=49)	33 (75)	16 (84.2)	ns	
Days of invasive mechanical ventilation (median, Q1-Q3)	17 (7-33)	18.5 (12-18)	ns	
Length of ICU stay (days; median, Q1-Q3)	16 (9-29.5)	19 (14-39)	ns	
Probable or possible pulmonary mold infection ^e^ (n=11)	10 (22.7)	1 (5.3)	p=0.094	
Renal graft loss (n=3)	2 (4.5)	1 (5.2)	ns	
Death (n=36)	25 (56.8)	11 (57.9)	ns	

ATG, Anti-thymocyte globulin; CMV, cytomegalovirus; COVID-19, Coronavirus disease 2019; ICU, Intensive care unit; SARS-CoV-2; mTOR, mechanistic target of rapamycin; SARS-CoV-2, severe acute respiratory syndrome coronavirus 2; SOTR, Solid organ transplant recipient.

a All received nebulized amphotericin-B.

b One patient received a simultaneous pancreas-kidney transplant.

c Tacrolimus + cyclosporine.

d High-dose corticosteroids for COVID-19 pneumonia was defined if the patient received a threshold of prednisone equivalent of 75mg/day.

e Ten COVID-19-associated pulmonary aspergillosis and one pulmonary mucormycosis.

ns, not significant (p>0.05).

Concerning adverse effects, only 1 of the 19 patients receiving nebulized amphotericin-B prophylaxis developed mild-to-moderate bronchospasm, and this did not require the interruption of prophylaxis. Finally, in-hospital mortality was comparable between groups.

## 4 Discussion and conclusion

In this multicenter observational study, antifungal prophylaxis with nebulized amphotericin-B among SOTRs admitted to ICU with COVID-19 showed a trend to lower the incidence of CAPA: 1 of 19 patients (5.3%) using this prophylaxis compared with 9 of 44 (20.5%) without prophylaxis. These results suggest that nebulized amphotericin-B might be effective in preventing CAPA in SOTRs. Our results are in line with two previous observational studies that showed a reduction in CAPA diagnosis in ICU-admitted COVID-19 patients with posaconazole (Hatzl et al., 2021) and nebulized amphotericin-B (Van Ackerbroeck et al., 2021) prophylaxis. However, while CAPA is associated with a higher mortality in other observational studies (Bartoletti et al., 2021; Dupont et al., 2021; Hatzl et al., 2021; Xu et al., 2021; Gangneux et al., 2022; Prattes et al., 2022), we found no differences in mortality nor length of ICU stay. Similar to a study of how antifungal prophylaxis affected CAPA, we also observed no survival benefit with inhaled amphotericin-B (Hatzl et al., 2021). The small sample size and number of CAPA events precludes the detection of meaningful clinical outcomes.

Our study has several limitations. Adverse events were not recorded prospectively in a standardized format and intolerance may therefore be underreported. Nonetheless, no discontinuation of prophylaxis due to side effects was recorded. Antifungal prophylaxis assignment was not randomized, meaning that baseline characteristics were not completely balanced between the two study groups. However, most known risk factors for aspergillosis were more common in the prophylaxis group (e.g., prior cytomegalovirus viremia, high-dose corticosteroids for COVID-19 and preexisting lung disease). In addition, none of the CAPA cases could be classified as proven, underlying the difficulties in diagnosing CAPA in ICU admitted patients. Despite the multicenter study design, the study population is small and included few CAPA events, and this limits both the reproducibility and the generalizability of the results of the study itself. However, the observed CAPA prevalence in this study might have been underestimated in the absence of standardized screening. Given that the range of CAPA prevalence is wide in other studies, it may not be possible to extrapolate our results to other geographic regions.

Despite the limitations of our research, targeted antifungal prophylaxis with nebulized amphotericin-B may still be justified for SOTRs admitted to ICU with COVID-19. First, patients admitted to ICU with COVID-19 have an estimated prevalence of 18%–19% for CAPA (Bartoletti et al., 2021; Dupont et al., 2021; Xu et al., 2021; Gangneux et al., 2022; Prattes et al., 2022), which could be higher in SOTRs given their baseline immunosuppression. Second, not only do the radiological features of pulmonary aspergillosis overlap with those of COVID-19 (Koehler et al., 2021) but also most diagnostic methods have lower sensitivities in non-hematological patients (Ullmann et al., 2018). Third, Aspergillus spp. treatment increases the risk of drug-drug interactions and toxicity among SOTRs (Luong et al., 2012; Lempers et al., 2015).

In conclusion, SOTRs admitted to ICU with respiratory failure due to SARS-CoV-2 infection have an elevated risk of CAPA. Nebulized amphotericin B represents an attractive choice for the prevention of CAPA in this high-risk population, benefiting from being effective and safe, while lacking drug-drug interactions (Rijnders et al., 2008; Paniagua Martin et al., 2010; Peghin et al., 2016; Van Ackerbroeck et al., 2021; Huggins et al., 2022). A randomized clinical trial to confirm these findings is warranted.

## Data availability statement

The raw data supporting the conclusions of this article will be made available by the authors, without undue reservation.

## Ethics statement

The studies involving human participants were reviewed and approved by Clinical Research Ethics Committee of the Bellvitge University Hospital. Written informed consent for participation was not required for this study in accordance with the national legislation and the institutional requirements.

## Author contributions

AR, MB, NS, JC conceived of the presented idea, AR, MB, VD-G, IC, AF, LL, JG-C, FO, MC-L, OL, EM-A, XN-C, DMG, JL participated in the data collection, AR, NS performed the data analysis and interpretation, AR, JC and NS drafted the article, MB, OL, JC and NS provided critical revision. All authors contributed to the article and approved the submitted version.
