# Supplementary material for: Antifungal prophylaxis with nebulized amphotericin-B in solid-organ transplant recipients with severe COVID-19: a retrospective observational study
Source: Front Cell Infect Microbiol. 2023 Apr 27;13:1165236. doi: 10.3389/fcimb.2023.1165236 (PMC10174318; doi:10.3389/fcimb.2023.1165236)
Supplement: Supplementary file 1 [file DataSheet_1.docx]

**Table S1. ECMM/ISHAM criteria used to diagnose CAPA.**

No proven, nine probable and one possible pulmonary aspergillosis were diagnosed according to the European Confederation of Medical Mycology (ECMM)/International Society for Human and Animal Mycoses (ISHAM) criteria.

Probable pulmonary forms according to the ECMM/ISHAM criteria:

All probable CAPA cases, had new or worsening infiltrates, one had new onset multiple cavitating lesions plus the following mycological evidence.

| Mycological evidence |
| --- |
| Two positive serum galactomannan antigen (≥0.5) and positive sputum culture |
| Positive serum galactomannan antigen (≥ 0.5) |
| ß-D-glucan (≥80 ng/L) in two consecutive serum samples; positive BAL galactomannan antigen (≥1) and positive bronchial brush culture |
| Positive BAL galactomannan antigen (≥1) and positive sputum culture |
| Positive BAL galactomannan antigen (≥1) and positive sputum culture |
| Positive (BAL) galactomannan antigen (≥1) and positive bronchial brush culture |
| Positive BAL galactomannan antigen (≥1) and positive PCR in BAL fluid (<36 cycles) |
| Positive BAL culture and positive PCR in BAL fluid (<36 cycles) |
| Positive culture in two bronchial brush and one sputum culture |

Possible pulmonary forms according to the ECCM/ISHAM criteria:

Worsening infiltrates on chest X-ray plus the following mycological evidence:

| Mycological evidence |
| --- |
| Positive cultures in more than two separated bronchial brush samples |

**Table S2**. **Comparison between SOTRs with COVID-19 admitted to ICU without and with possible or probable pulmonary aspergillosis**.

| Variable | Aspergillosis diagnosis | | p |
| --- | --- | --- | --- |
|  | Without, n=53 (%) | With, n=10 (%) |  |
| **Patients characteristics** | | | |
| Age (mean; SD) | 62.4 (11.6) | 60.9 (12.9) | ns |
| Female (n=16) | 13 (24.5) | 3 (30) | ns |
| Active smoker (n=2) | 2 (3.8) | 0 (0) | ns |
| High blood pressure (n=54) | 44 (83) | 10 (10) | ns |
| CRF (creatinine > 136 μmol/L) (n=41) | 34 (64.2) | 7 (70) | ns |
| Diabetes mellitus (n=26) | 21 (39.6) | 5 (50) | ns |
| Preexisting heart diseases (n=25) | 20 (37.7) | 5 (50) | ns |
| BMI >30 (n=15) | 12 (22.6) | 3 (30) | ns |
| Preexisting lung diseases (n=13) | 11 (20.8) | 2 (20) | ns |
| Chronic hepatopathy (n=4) | 3 (5.7) | 1 (10) | ns |
| Kidney transplant ^‡^ (n=50) | 43 (81.1) | 7 (70) | ns |
| Lung transplant (n=6) | 6 (11.3) | 0 (0) | ns |
| Heart transplant (n=4) | 2 (3.8) | 2 (20) | ns |
| Liver transplant (n=3) | 2 (3.8) | 1 (10) | ns |
| Recent SOTR (<6 months) (n=16) | 14 (26.4) | 2 (20) | ns |
| Hepatitis C infection (n=2) | 1 (1.9) | 1 (10) | ns |
| CMV viremia (<3 months) (n=7) | 7 (13.2) | 0 (0) | ns |
| Transplant rejection (<3 months) (n=6) | 6 (11.3) | 0 (0) | ns |
| Corticosteroid bolus (<3 months) (n=9) | 9 (17) | 0 (0) | ns |
| Monoclonal antibodies for rejection (<3 months) (n=6) | 5 (9.4) | 1 (10) | ns |
| Thymoglobulin (<6 months) (n=2) | 2 (3.8) | 0 (0) | ns |
| Vaccinated for SARS-CoV-2 (n=30) | 22 (41.5) | 8 (80) | p=0.025 |
| ***Immunosuppressant medications at admission*** | | | |
| Cyclosporine (n=2) | 1 (1.9) | 1 (10) | ns |
| Tacrolimus (n=57) | 48 (90.6) | 9 (90) | ns |
| Sirolimus (n=2) | 2 (3.8) | 0 (0) | ns |
| Everolimus (n=2) | 2 (3.8) | 0 (0) | ns |
| Mycophenolate Mofetil (n=54) | 45 (84.9) | 9 (90) | ns |
| ***Immunosuppressant medications at ICU admission*** | | | |
| Calcineurin inhibitors ^¶^ (n=42) | 35 (66) | 7 (70) | ns |
| mTOR inhibitor (n=2) | 2 (3.8) | 0 (0) | ns |
| Mycophenolate Mofetil (n=9) | 8 (15.1) | 1 (10) | ns |
| ***Treatment*** | | | |
| Tocilizumab (n=16) | 11 (20.8) | 5 (50) | p=0.051 |
| Remdesivir (n=6) | 6 (11.3) | 0 (0) | ns |
| Corticosteroids (n=61) | 52 (98.1) | 9 (90) | ns |
| Inhaled amphotericin B prophylaxis (n=19) | 18 (34) | 1 (10) | p=0.13 |
| ***Clinical course*** | | | |
| Invasive mechanical ventilation (n=49) | 40 (75.5) | 9 (90) | ns |
| Days of IMV, median (Q1-Q3) | 17 (8-28) | 16 (5.3-43) | ns |
| Length of ICU stay in days, median (Q1-Q3) | 17 (10-30) | 15 (9-48) | ns |
| Renal graft loss (n=3) | 2 (3.8) | 1 (10) | ns |
| Death (n=36) | 30 (56.6) | 6 (60) | ns |

‡ One patient received a simultaneous pancreas-kidney transplant. ^¶^ Tacrolimus and cyclosporine

*Abbreviations:* BMI, body mass index; CMV, cytomegalovirus; COVID-19, Coronavirus disease 2019; CRF, Chronic renal failure; ICU, Intensive care unit; IMV, invasive mechanical ventilation; IQR, interquartile range; mTOR, mechanistic target of rapamycin; SARS-CoV-2, severe acute respiratory syndrome coronavirus 2; SOTR, Solid organ transplant recipient.

**Rationale behind the nebulized amphotericin-B dosing regimen:**

Nebulised AMB is standard prophylaxis in lung transplants at the Hospital Universitari Vall d’Hebron, all lung transplant receive 25mg of nebulized L-AMB three days a week the first 60 days, with lower doses thereafter. All lung transplant recipients receive nebulized AMB prophylaxis for life and when admitted the regimen is changed to higher doses (L-AMB 25mg three days a week or desoxycholate 6mg/8h when intubated). Universal antifungal prophylaxis with nebulised AMB during hospitalization (using the same regimen) is also standard care at the Hospital de Bellvitge for heart transplant recipients from 2015. Previous pharmacokinetics and safety studies have been performed in lung transplant recipients^1^. The use of nebulized AMB for SOTRs admitted to ICU for severe COVID-19 was not protocoled. After witnessing several CAPA cases, the infectious diseases consultant encouraged the use of prophylaxis with nebulized amphotericin-B in COVID-19 ICU admitted SOTRs, although the final decision on initiating prophylaxis remained with the treating intensivist.

**References**:

1. Monforte V, Ussetti P, López R, et al. Nebulized liposomal amphotericin B prophylaxis for Aspergillus infection in lung transplantation: pharmacokinetics and safety. J Heart Lung Transplant. 2009;28(2):170-175. doi:10.1016/j.healun.2008.11.004
